# Information equilibria, subsystem entanglement, and dynamics of the overall entropic descriptors of molecular electronic structure

**DOI:** 10.1007/s00894-018-3699-3

**Published:** 2018-07-19

**Authors:** Roman F. Nalewajski

**Affiliations:** Department of Theoretical Chemistry, Jagiellonian University, Gronostajowa 2, 30-387 Cracow, Poland

**Keywords:** Entropic principles/equilibria, Information theory, Probability/phase dynamics, Quantum entropy, Resultant entropy/information, Subsystem entanglement

## Abstract

Overall descriptors of the information (determinicity) and entropy (uncertainty) content of complex molecular states are reexamined. These resultant concepts combine the classical (probability) contributions of Fisher and Shannon, and the relevant nonclassical supplements due to the state phase/current. The information-theoretic principles determining equilibria in molecules and their fragments are explored and the nonadditive part of the global entropy is advocated as a descriptor of the classical index of the quantum entanglement of molecular subsystems. Affinities associated with the probability and phase fluxes are identified and the criterion of vanishing overall information-source is shown to identify the system stationary electronic states. The production of resultant density of the gradient-information is expressed in terms of the conjugate affinities (forces, perturbations) and fluxes (currents, responses). The Schrödinger dynamics of probability and phase components of molecular electronic states is used to determine the temporal evolution of the overall gradient information and complex entropy. The global sources of the resultant information/entropy descriptors are shown to be of purely nonclassical origin, thus identically vanishing in real electronic states, e.g., the nondegenerate ground state of a molecule.

## Introduction

The electronic structure of molecules is embodied in their quantum states generating both the system particle density and current distributions. The continuity relation for the state probability density, which relates these two structural aspects of molecular wavefunctions, implies that the density dynamics is determined by the current’s divergence. To paraphrase Prigogine [1], while the electron density determines a static facet of “being”, the probability current reflects the state dynamic aspect of “becoming”. A general electronic wavefunction is a complex entity characterized by both its modulus and phase components. The square of the former determines the particle probability distribution marking the structure of “being”, while the gradient of the latter generates the state current density reflecting the structure of “becoming”. These two structural manifestations give rise to the associated classical and nonclassical contributions in the resultant measures of the information/entropy content in complex electronic states [2]. Both the probability and phase/current distributions carry *partial* information contributions to the *resultant* entropic content of the underlying quantum state of a molecule.

In entropic theories of molecular electronic structure, e.g., [2–5], one thus requires an appropriate quantum generalization [2] of the familiar classical descriptors of information theory (IT) [6–13], of the information content in the probability distribution. The quantum extensions [2] of the Fisher (gradient) [6, 7] and Shannon (global) [8, 9] measures, appropriate for *complex* amplitudes (wavefunctions) of molecular quantum mechanics (QM), combine the partial contributions due to probability (wavefunction modulus) and current (wavefunction phase) degrees-of-freedom. In the position representation the electron probability distribution *p*(***r***) alone generates the state *classical* amount of information, i.e., the information received from outcomes of *incoherent* (*phase*-unrelated) local events, outcomes of measurements of the particle position ***r***. Their *nonclassical* complements in the *resultant* entropy/information measures, describing the coherent (*phase*-related) local events, generate the corresponding *coherence* entropy/information supplements [2, 14–20] due to the state phase *ϕ*(***r***) or the current density ***j***(***r***) ∝ ∇*ϕ*(***r***). Similar generalized descriptors of both the overall information content and entropy-deficiency (information-distance) [10, 11] can be introduced in the momentum space [2, 21].

The classical IT [6–13], an important branch of the applied probability theory, has already provided new insights into molecular electronic structure and generated useful descriptors of atoms in molecules, reactivity preferences and patterns of chemical bonds, e.g., [2–5]. The classical information terms are conceptually related to modern density functional theory (DFT) [22–24]. They probe the entropic content of incoherent localization events, the outcomes of experiments measuring the particle position, while their nonclassical companions provide the information supplement due to the *phase*-coherence between such local events, which is inherent in general wavefunctions of QM. The familiar average information/entropy measures of Fisher and Shannon reflect only the information/entropy content in the system wavefunction due to the probability distribution; thus, failing to distinguish states exhibiting the same electron density but different current compositions.

Therefore, in the *quantum* IT (QIT) description of the phase equilibria in molecular systems and their constituent fragments [2, 16–21, 25–29] one has to unite both the probability and phase/current aspects of the system quantum states, in order to fully characterize the overall information content in molecular wavefunctions, the equilibrium states of both the system as a whole and its constituent parts, a degree of the quantum entanglement (mutual bonding status) of subsystems, or the electron diffusion processes [28]. The recently introduced *resultant* IT descriptors combine the *classical* probability contributions with their respective *nonclassical* supplements due to the state phase/current. The densities of nonclassical information/entropy terms exhibit the same mutual relations as their classical analogs and they introduce the nonvanishing source terms into their respective continuity relations [2, 26]. They have been successfully used to establish the phase equilibria in molecules, and to distinguish the mutually bonded (*phase*-related, “entangled”) status of molecular fragments and reactants from its nonbonded (*phase*-unrelated, “disentangled”) analog [2, 27–31].

The complex global entropy [2, 25], the expectation value of a *non*-Hermitian operator, generates the probability and phase contributions in the resultant measure as its real and imaginary parts. This *two*-component (“vector”) extension satisfies the requirement that a classical dependence between densities-per-electron of the ordinary Shannon and Fisher entropy/information measures also covers the interrelation between their nonclassical supplements. The *phase*-dependent concept of complex entropy will be related to the Shannon entropy of information theory and von Neumann’s entropy in density matrix. The gradient entropy (*indeterminicity*-information) analog of the resultant Fisher (*determinicity*-information) descriptor has also been conjectured [2]. It combines the classical Fisher information with the negative nonclassical phase/current supplement. Indeed, the presence of a finite current introduces additional structure, “order” element, thus increasing the state information (determinicity) content and decreasing its entropy (uncertainty) property of electronic “disorder”.

Generalized information principles, formulated in terms of resultant IT descriptors, identify extrema of the global and gradient entropies, which mark the *phase*-equilibria in molecules [14–20]. Such states exhibit a “thermodynamic” *phase*-shift related to the logarithm of probability density. The phase transformation defining the system equilibrium wavefunction affects both the local probability source and the net entropy production in the associated continuity equations [2, 18, 26]. Similar equilibria can be determined in the “entangled” (bonded) and “disentangled” (nonbonded) states of molecular fragments [2, 27–31].

In this work relations between densities of the the complex entropy and resultant information measures will be reexamined and the *phase* and *information* equilibria, marking extrema of the resultant entropy and information measures, respectively, will be explored. The nonadditive part of the global entropy in the subsystem resolution will be advocated as a classical information descriptor of the quantum entanglement of the partition molecular fragments. The “vector” character of complex entropy density raises the natural question of what “scalar” function of its real (probability) and imaginary (phase) parts determines the information principle for establishing molecular phase-equilibria. This information quantity will be identified as the resultant gradient entropy.

The source term in the continuity relation for the overall gradient information will be examined and the underlying state affinities (“forces”) and fluxes (“responses”) will be identified; the equilibrium criterion of the vanishing information production will be shown to determine the stationary states of molecular QM. The time evolution of the QIT entropy/information measures will be explored using the the dynamical equations for the probability and phase components of electronic wavefunctions implied by the molecular Schrödinger Eq. (SE). The time dependence of the resultant information/entropy will be expressed in terms of the state probability and phase degrees-of-freedom, and the nonclassical origins of these derivatives will be revealed.

## Probability and phase components of electronic states

Let us consider a single electron (*N* = 1) at time *t*_0_ = 0 in state |*ψ*(*t*_0_)〉 ≡ |*ψ*(0)〉 ≡ |*ψ*〉 described by the complex wave function in *position*-representation,

1 $$ \psi \left(\boldsymbol{r},0\right)=\left\langle \boldsymbol{r}|\psi (0)\right\rangle =R\left(\boldsymbol{r}\right)\ \exp \left[\mathrm{i}\phi \left(\boldsymbol{r}\right)\right]\equiv \psi \left(\boldsymbol{r}\right), $$

where *R*(***r***) and *ϕ*(***r***) stand for its modulus and phase parts. It determines the probability distribution,

2 $$ p\left(\boldsymbol{r},0\right)=\left\langle \psi \left.(0)\right|\boldsymbol{r}\right\rangle \left\langle \left.\boldsymbol{r}\right|\psi (0)\right\rangle \equiv \left\langle \left.\psi \right|\widehat{\uprho}\left.\left(\boldsymbol{r}\right)\right|\psi \right\rangle =\boldsymbol{\psi} {\left(\boldsymbol{r}\right)}^{\ast}\boldsymbol{\psi} \left(\boldsymbol{r}\right)=R{\left(\boldsymbol{r}\right)}^2\equiv p\left(\boldsymbol{r}\right), $$

and its current

3 $$ {\displaystyle \begin{array}{l}\boldsymbol{j}\left(\boldsymbol{r},0\right)={(2m)}^{-1}\left\langle \psi (0)\left|\left\{\uprho \left(\boldsymbol{r}\right)\;\mathbf{p}+\mathbf{p}\;\uprho \left(\boldsymbol{r}\right)\right\}\right|\psi (0)\right\rangle \equiv \left\langle \psi \left|\widehat{\mathbf{j}}\left(\boldsymbol{r}\right)\right|\psi \right\rangle \equiv \boldsymbol{j}\left(\boldsymbol{r}\right)\\ {}=\left[\hbar /\left(2m\mathrm{i}\right)\right]\left[\psi {\left(\boldsymbol{r}\right)}^{\ast}\nabla \psi \left(\boldsymbol{r}\right)-\psi \left(\boldsymbol{r}\right)\nabla \psi {\left(\boldsymbol{r}\right)}^{\ast}\right]=\left(\hbar /m\right)\operatorname{Im}\left[\psi {\left(\boldsymbol{r}\right)}^{\ast}\nabla \psi \left(\boldsymbol{r}\right)\right]\\ {}=\left(\hbar /m\right)p\left(\boldsymbol{r}\right)\nabla \phi \left(\boldsymbol{r}\right)\equiv p\left(\boldsymbol{r}\right)\boldsymbol{V}\left(\boldsymbol{r}\right);\end{array}} $$

here the momentum operator $\widehat{\mathbf{p}}$ is defined by its action on the wavefunction 〈***r***|$\widehat{\mathbf{p}}\psi$〉 = −i*ħ*∇*ψ*(***r***), and the average velocity ***V***(***r***) of the probability fluid, measuring the current-per-particle, reflects the state phase-gradient:

4 $$ \boldsymbol{V}\left(\boldsymbol{r}\right)=\boldsymbol{j}\left(\boldsymbol{r}\right)/p\left(\boldsymbol{r}\right)=\left(\hbar /m\right)\ \nabla \phi \left(\boldsymbol{r}\right). $$

The wavefunction modulus, the classical amplitude of the particle probability density, and the state phase or its gradient, determining the effective velocity and probability flux, thus constitute two fundamental degrees-of-freedom in the full quantum IT treatment of electronic states: *ψ* ⇔ (*R*, *ϕ*) ⇔ (*p*, ***j***).

One envisages the electron moving in the external potential *v*(***r***) due to the “frozen” nuclei of the molecule, described by the electronic Hamiltonian

5 $$ \widehat{\mathrm{H}}\left(\boldsymbol{r}\right)=-\left({\hbar}^2/2m\right){\nabla}^2+v\left(\boldsymbol{r}\right)\equiv \widehat{\mathrm{T}}\left(\boldsymbol{r}\right)+v\left(\boldsymbol{r}\right), $$

where $\widehat{\mathrm{T}}\left(\boldsymbol{r}\right)$ denotes its kinetic part. The quantum dynamics of a general electronic state |*ψ*(*t*)〉, giving rise to the associated wavefunction

6 $$ \psi \left(\boldsymbol{r},t\right)=\left\langle \boldsymbol{r}|\psi (t)\right\rangle =R\left(\boldsymbol{r},t\right)\ \exp \left[\mathrm{i}\phi \left(\boldsymbol{r},t\right)\right]\equiv R(t)\ \exp \left[\mathrm{i}\phi (t)\right]\equiv \psi (t), $$

is generated by SE,

7 $$ \partial \psi (t)/\partial t={\left( i\mathit{\hbar}\right)}^{-1}\widehat{\mathrm{H}}\psi (t), $$

which also determines temporal evolutions of the state two physical components: the instantaneous probability density *p*(***r***, *t*) = |*ψ*(***r***, *t*)|^2^ = *R*(***r***, *t*)^2^ ≡ *p*(*t*), and the state phase *ϕ*(***r***, *t*) ≡ *ϕ*(*t*). The total time derivative of the former expresses the sourceless continuity relation for the probability distribution,

8a $$ {\displaystyle \begin{array}{l}{\sigma}_p\left(\boldsymbol{r},t\right)\equiv dp\left(\boldsymbol{r},t\right)/ dt=\partial p\left(\boldsymbol{r},t\right)/\partial t+\nabla \cdot \boldsymbol{j}\left(\boldsymbol{r},t\right)\\ {}=\partial p\left(\boldsymbol{r},t\right)/\partial t+\boldsymbol{V}\left(\boldsymbol{r},t\right)\cdot \nabla p\left(\boldsymbol{r},t\right)+p\left(\boldsymbol{r},t\right)\nabla \cdot \boldsymbol{V}\left(\boldsymbol{r},t\right)\\ {}=\partial p\left(\boldsymbol{r},t\right)/\partial t+\left(d\boldsymbol{r}/ dt\right)\cdot \left[\partial p\left(\boldsymbol{r},t\right)/\partial \boldsymbol{r}\right]=\partial p\left(\boldsymbol{r},t\right)/\partial t+\boldsymbol{V}\left(\boldsymbol{r},t\right)\cdot \nabla p\left(\boldsymbol{r},t\right)=0\kern1.75em \mathrm{or}\kern2em \end{array}} $$

8b $$ {\displaystyle \begin{array}{c}\partial p\left(\boldsymbol{r},t\right)/\partial t=-\mathit{\nabla}\cdot \boldsymbol{j}\left(\boldsymbol{r},t\right)=-\left(\hbar /2m\mathrm{i}\right)\left[\psi {\left(\boldsymbol{r},t\right)}^{\ast}\Delta \psi \left(\boldsymbol{r},t\right)-\psi \left(\boldsymbol{r},t\right)\Delta \psi {\left(\boldsymbol{r},t\right)}^{\ast}\right]\\ {}=-\left(\hbar /m\right)\left[\mathit{\nabla \phi}\left(\boldsymbol{r},t\right)\cdot \mathit{\nabla p}\left(\boldsymbol{r},t\right)+p\left(\boldsymbol{r},t\right){\mathit{\nabla}}^2\phi \left(\boldsymbol{r},t\right)\right].\end{array}} $$

The total derivative,

9 $$ dp\left(\boldsymbol{r},t\right)/ dt=\partial p\left(\boldsymbol{r},t\right)/\partial t+d\boldsymbol{r}/ dt\cdot \partial p\left(\boldsymbol{r},t\right)/\partial \boldsymbol{r}=\partial p\left(\boldsymbol{r},t\right)/\partial t+\boldsymbol{V}\left(\boldsymbol{r},t\right)\cdot \nabla p\left(\boldsymbol{r},t\right), $$

determining the local probability “source” *σ*_*p*_(***r***, *t*), has been interpreted above as the time rate of change in a moving infinitesimal volume element of the probability fluid, while the partial derivative ∂*p*(***r***, *t*)/∂*t* represents the corresponding rate at the fixed point in space. The probability continuity thus implies:

10 $$ dp\left(\boldsymbol{r},t\right)/ dt+p\left(\boldsymbol{r},t\right)\ \nabla \cdot \boldsymbol{V}\left(\boldsymbol{r},t\right)=0. $$

Thus, the vanishing probability source of Eq. (8a) also implies the vanishing divergence of the velocity field: ∇⋅***V***(***r***, *t*) = 0. This probability continuity equation also determines the dynamics of the state modulus component:

11 $$ \mathit{\partial R}\left(\boldsymbol{r},t\right)/\partial t=-\left(\hbar /m\right)\ \left[\nabla \phi \left(\boldsymbol{r},t\right)\cdot \nabla R\left(\boldsymbol{r},t\right)+\left[R\left(\boldsymbol{r},t\right)/2\right]\ \Delta \phi \left(\boldsymbol{r},t\right)\right]. $$

The particle effective velocity also determines the current concept associated with the state phase: ***J***(***r***, *t*) = *ϕ*(***r***, *t*) ***V***(***r***, *t*). The scalar field *ϕ*(***r***, *t*) and its conjugate current density ***J***(***r***, *t*) determine a nonvanishing phase source [2] in the associated continuity equation:

12 $$ {\displaystyle \begin{array}{l}{\sigma}_{\phi}\left(\boldsymbol{r},t\right)\equiv d\phi \left(\boldsymbol{r},t\right)/ dt=\partial \phi \left(\boldsymbol{r},t\right)/\partial t+\mathit{\nabla}\cdot \boldsymbol{J}\left(\boldsymbol{r},t\right)=\partial \phi \left(\boldsymbol{r},t\right)/\partial t+\boldsymbol{V}\left(\boldsymbol{r},t\right)\cdot \mathit{\nabla \phi}\left(\boldsymbol{r},t\right)\ne 0\kern1.25em \mathrm{or}\\ {}\partial \phi \left(\boldsymbol{r},t\right)/\partial t-{\sigma}_{\phi}\left(\boldsymbol{r},t\right)=-\mathit{\nabla}\cdot \boldsymbol{J}\left(\boldsymbol{r},t\right)=-\left(\hbar /m\right)\left\{{\left[\mathit{\nabla \phi}\left(\boldsymbol{r},t\right)\right]}^2+\phi \left(\boldsymbol{r},t\right)\Delta \phi \left(\boldsymbol{r},t\right)\right\}.\end{array}} $$

The phase dynamics from SE,

13 $$ \partial \phi /\partial t=\left[\hbar /(2m)\right]\ \left[{R}^{-1}\Delta R-{\left(\nabla \phi \right)}^2\right]-v/\hbar, $$

finally identifies the phase source:

14 $$ {\sigma}_{\phi }=\left[\hbar /(2m)\right]\ \left[{R}^{-1}\Delta R+{\left(\nabla \phi \right)}^2\right]-v/\hbar . $$

As an illustration consider the stationary wavefunction corresponding to energy *E*_*s*_,

15 $$ {\displaystyle \begin{array}{ll}{\psi}_s\left(\boldsymbol{r},t\right)={R}_s\left(\boldsymbol{r}\right)\ \exp \left[\mathrm{i}{\phi}_s(t)\right],& {\phi}_s(t)=-\left({E}_s/\hbar \right)t=-{\omega}_st;\\ {}{p}_s\left(\boldsymbol{r},t\right)={R}_s{\left(\boldsymbol{r}\right)}^2={p}_s\left(\boldsymbol{r}\right),& {\boldsymbol{j}}_s\left(\boldsymbol{r},t\right)={\boldsymbol{V}}_s\left(\boldsymbol{r},t\right)=\boldsymbol{0},\end{array}} $$

representing an eigenstate of the Hamiltonian:

16 $$ \widehat{\mathrm{H}}\left(\boldsymbol{r}\right)\kern0.24em {R}_s\left(\boldsymbol{r}\right)=-\left({\hbar}^2/2m\right)\Delta {R}_s\left(\boldsymbol{r}\right)+v\left(\boldsymbol{r}\right){R}_s\left(\boldsymbol{r}\right)={E}_s{R}_s\left(\boldsymbol{r}\right). $$

The phase dynamics of Eq. (13) then recovers the stationary SE and identifies a constant phase source:

17 $$ \left(\partial {\phi}_s/\partial t\right)={\sigma}_{\phi }=\left[\hbar /(2m)\right]\ \left({R_s}^{-1}\Delta {R}_s\right)-v=-{\omega}_s= const. $$

## Resultant information/entropy concepts and uncertainty principle

At a given instant *t* = *t*_0_ the average Fisher [6] measure of the classical gradient information for locality events, contained in the molecular probability density *p*(***r***) = *R*(***r***)^2^, is reminiscent of von Weizsäcker’s [32] inhomogeneity correction to the kinetic energy functional in Thomas-Fermi theory,18here ***ℐ***_*p*_(***r***) = *p*(***r***) *I*_*p*_(***r***) denotes the functional density and *I*_*p*_(***r***) stands for the associated density-per-electron. The amplitude form *I*[*R*] reveals that this classical descriptor measures the average length of the modulus gradient ∇*R*. This classical, probability descriptor characterizes an effective “narrowness” of *p*(***r***), i.e., a degree of determinicity of the particle position.

The classical Shannon (S) [8] descriptor of the global entropy in *p*(***r***),19similarly reflects the distribution “spread” (uncertainty), i.e., a degree of the position indeterminacy. It also provides the amount of information received, when this uncertainty is removed by an appropriate particle-localization experiment: *I*^S^[*p*] ≡ *S*[*p*]. The densities-per-electron of these complementary information and entropy functionals are seen to satisfy the classical relation:

20 $$ {I}_p\left(\boldsymbol{r}\right)={\left[\nabla {S}_p\left(\boldsymbol{r}\right)\right]}^2 $$

These probability functionals of the *classical* information/entropy content generalize naturally into the corresponding *resultant* quantum descriptors combining the probability and phase/current contributions to the overall entropy/information descriptors of the electronic state |*ψ*〉 [2, 14–20]. Such generalized concepts are applicable to complex wavefunctions of molecular QM. They are defined as average values of the associated operators: the Hermitian operator of the gradient information [33] related to the kinetic energy operator $\widehat{\mathrm{T}}\left(\boldsymbol{r}\right)$,

21 $$ \widehat{\mathrm{I}}\left(\boldsymbol{r}\right)=-4\Delta ={\left(2 i\mathit{\nabla}\right)}^2=\left(8m/{\hbar}^2\right)\widehat{\mathrm{T}}\left(\boldsymbol{r}\right), $$

and the non-Hermitian (multiplicative) operator of the state complex entropy [25],

22 $$ {\widehat{\mathrm{S}}}_{\psi}\left(\boldsymbol{r},t\right)=-2\ln \psi \left(\boldsymbol{r},t\right)\equiv {S}_{\psi}\left(\boldsymbol{r},t\right)=-\ln p\left(\boldsymbol{r},t\right)-2\mathrm{i}\phi \left(\boldsymbol{r},t\right). $$

Therefore, their quantum expectation values in state |*ψ*〉 give rise to real and complex average IT descriptors, respectively.

The overall gradient *infomation* combines the classical (probability) and nonclassical (phase/current) contributions:23This resultant (real) *gradient information* is proportional to the state average kinetic energy:

$$ T\left[\psi \right]=\left\langle \psi |\widehat{\mathrm{T}}|\psi \right\rangle =\left({\hbar}^2/8m\right)I\left[\psi \right]. $$

The resultant complex (“vector”) measure of the state *global entropy* is similarly determined by its real (classical) and imaginary (nonclassical) contributions:24The densities-per-electron of these functionals,

25 $$ {\displaystyle \begin{array}{l}{I}_{\psi}\left(\boldsymbol{r}\right)={I}_p\left(\boldsymbol{r}\right)+{\left[2\mathit{\nabla \phi}\left(\boldsymbol{r}\right)\right]}^2\equiv {I}_p\left(\boldsymbol{r}\right)+{I}_{\phi}\left(\boldsymbol{r}\right)\\ {}={I}_p\left(\boldsymbol{r}\right)+{\left\{\left(2m/\hbar \right)\boldsymbol{j}\left(\boldsymbol{r}\right)/p\left(\boldsymbol{r}\right)\right\}}^2={I}_p\left(\boldsymbol{r}\right)+{\left[\left(2m/\hbar \right)\boldsymbol{V}\left(\boldsymbol{r}\right)\right]}^2\equiv {I}_p\left(\boldsymbol{r}\right)+{I}_{\boldsymbol{j}}\left(\boldsymbol{r}\right)\end{array}} $$

and

26 $$ {H}_{\psi}\left(\boldsymbol{r}\right)={S}_p\left(\boldsymbol{r}\right)-2\mathrm{i}\phi \left(\boldsymbol{r}\right)\equiv {S}_p\left(\boldsymbol{r}\right)+\mathrm{i}{S}_{\phi}\left(\boldsymbol{r}\right), $$

now satisfy the complex generalized relation

27 $$ {I}_{\psi}\left(\boldsymbol{r}\right)={\left|\nabla {H}_{\psi}\left(\boldsymbol{r}\right)\right|}^2={\left[\nabla {S}_p\left(\boldsymbol{r}\right)\right]}^2+{\left[\nabla {S}_{\phi}\left(\boldsymbol{r}\right)\right]}^2. $$

One also introduces the resultant *gradient entropy* [2], the state *local* uncertainty descriptor (indeterminicity-information),28exhibiting a nonpositive phase supplement *M*_*ϕ*_(***r***) = − *I*[*ϕ*] in its overall density-per-electron: *M*_*ψ*_(***r***) ≡ *M*_*p*_(***r***) + *M*_*ϕ*_(***r***) = *I*_*p*_(***r***) − *I*_*ϕ*_(***r***). Indeed, the presence of a finite probability current ***j***(***r***) ≠ ***0***, generated by the local phase *ϕ*(***r***) > 0, implies an additional “structure” element of the current distribution in the electronic state, thus increasing its resultant *information* (“order”) density, *I*_*ϕ*_(***r***) > 0, and lowering the associated *entropy* (“disorder”) contribution: *M*_*ϕ*_(***r***) < 0.

The global entropy of probability distribution has also been generalized in the resultant “scalar” measure of the uncertainty content in the specified quantum state *ψ* [2]:29It represents the expectation value of the Hermitian operator of the scalar measure of resultant global entropy,30and combines the classical contribution *S*[*p*] ≥ 0 of Shannon with its nonclassical supplement *S*[*ϕ*] = −2〈*ϕ*〉_*ψ*_ ≤ 0 reflecting the average phase in state |*ψ*〉: 〈*ϕ*〉_*ψ*_ = ∫*p*(***r***) *ϕ*(***r***) *d****r*** ≥ 0.

To summarize, the modulus (probability) and phase (current) components of electronic states are both accounted for in the resultant measures of the gradient or global descriptors of the information/entropy content in generally complex wavefunctions of molecular QM. These overall descriptors combine the familiar classical functionals of the system probability density and their nonclassical supplements due to the state phase or its current density. Their densities-per-electron satisfy classical relations linking the gradient and global descriptors, appropriately generalized to cover a complex character of electronic states. The Hermitian operator $\widehat{\mathrm{I}}\left(\boldsymbol{r}\right)$ gives rise to the real expectation value of the state content of resultant determinicity information *I*[*ψ*], related to the average kinetic energy *T*[*ψ*], while the non-Hermitian entropy operator ${\widehat{\mathrm{S}}}_{\psi}\left(\boldsymbol{r}\right)$ generates the complex average measure *H*[*ψ*] of the global uncertainty in *ψ*. The classical and nonclassical densities-per-electron of the resultant gradient information and the overall global entropy then separately obey the classical relations:

31 $$ {I}_p\left(\boldsymbol{r}\right)={\left[\mathit{\nabla}{H}_p\left(\boldsymbol{r}\right)\right]}^2\ \mathrm{and}\ {I}_{\phi}\left(\boldsymbol{r}\right)={\left[\mathit{\nabla}{H}_{\phi}\left(\boldsymbol{r}\right)\right]}^2={\left[ i\mathit{\nabla}{S}_{\phi}\left(\boldsymbol{r}\right)\right]}^2=-{\left[\mathit{\nabla}{S}_{\phi}\left(\boldsymbol{r}\right)\right]}^2. $$

The squared gradient of the classical and nonclassical components in the Shannon-type entropy density is thus seen to determine densities of the associated contributions to the resultant Fisher-type information:

32 $$ {\displaystyle \begin{array}{l}\widehat{\mathrm{I}}\left(\boldsymbol{r}\right)=\mathit{\nabla}{\widehat{\mathrm{S}}}_{\psi}\left(\boldsymbol{r}\right)\cdot \mathit{\nabla}{\widehat{\mathrm{S}}}_{\psi }{\left(\boldsymbol{r}\right)}^{\dagger }={\left|\mathit{\nabla}{\widehat{\mathrm{S}}}_{\psi}\left(\boldsymbol{r}\right)\right|}^2\\ {}={\left[\mathit{\nabla}\ln p\left(\boldsymbol{r}\right)\left]{}^2+\right[-2\mathit{\nabla \phi}\left(\boldsymbol{r}\right)\right]}^2={\left[\frac{\mathit{\nabla p}\left(\boldsymbol{r}\right)}{p\left(\boldsymbol{r}\right)}\right]}^2+4{\left[\mathit{\nabla \phi}\left(\boldsymbol{r}\right)\right]}^2\ge 0.\end{array}} $$

Therefore, the gradient of complex entropy can be regarded as the quantum amplitude of the resultant information content. In other words, *∇*${\widehat{\mathrm{S}}}_{\psi}\left(\boldsymbol{r}\right)$ appears as the “square root” of $\widehat{\mathrm{I}}\left(\boldsymbol{r}\right)$. This development is thus in spirit of the quadratic approach of Prigogine [1].

The (Hermitian) operator of the gradient entropy is seen to involve the sum of the ordinary squares of gradients of the operator components:

33 $$ {\widehat{\mathrm{M}}}_{\psi}\left(\boldsymbol{r}\right)={\left[\nabla {\widehat{\mathrm{S}}}_p\left(\boldsymbol{r}\right)\right]}^2+{\left[\nabla {\widehat{\mathrm{S}}}_{\phi}\left(\boldsymbol{r}\right)\right]}^2={\left[\mathrm{\nabla ln}p\left(\boldsymbol{r}\right)\right]}^2-4{\left[\nabla \phi \left(\boldsymbol{r}\right)\right]}^2. $$

This relation establishes the scalar information principle for determining the *phase*-equilibria [2, 14–20],

34 $$ {\psi}_{eq.}\left(\boldsymbol{r}\right)=R\left(\boldsymbol{r}\right)\ \exp \left\{\mathrm{i}\left[\phi \left(\boldsymbol{r}\right)+{\phi}_{eq.}\left(\boldsymbol{r}\right)\right]\right\}\equiv R\left(\boldsymbol{r}\right)\ \exp \left[\mathrm{i}{\varPhi}_{eq.}\left(\boldsymbol{r}\right)\right], $$

corresponding to “thermodynamic” phase shift *ϕ*_*eq.*_ (***r***) ≥ 0. More specifically, the extremum of *M*[*ψ*] with respect to ψ^*^, 〈*δψ*|${\widehat{\mathrm{M}}}_{\psi }$|*ψ*〉 = 0, gives the Euler equation

35 $$ {\left[\mathit{\nabla}\ln p\left(\boldsymbol{r}\right)\right]}^2=4{\left[\mathit{\nabla}{\phi}_{eq.}\left(\boldsymbol{r}\right)\right]}^2, $$

which identifies the equilibrium phase

36 $$ {\phi}_{eq.}\left(\boldsymbol{r}\right)=-\left(1/2\right)\ \ln p\left(\boldsymbol{r}\right)\ge 0. $$

The same optimum solution follows from the extremum rule for *S*[*ψ*], 〈*δψ*||*ψ*〉 = 0, or37It should be observed, however, that the associated extremum principle for the resultant gradient information *I*[*ψ*], 〈*δψ*|$\widehat{\mathrm{I}}$|*ψ*〉 = 0, predicts a pure-imaginary optimum phase *ϕ*_*opt.*_(***r***) = i*ϕ*_*eq.*_(***r***).

It is also of interest to examine how the familiar Heisenberg (“indeterminicity”) inequality for the product of squared dispersions in the particle position ***r*** and momentum ***p***,

$$ {\left[{\left\langle \delta \boldsymbol{r}\right\rangle}_{\psi}\right]}^2{\left[{\left\langle \delta \boldsymbol{p}\right\rangle}_{\psi}\right]}^2>{\hbar}^2/4,\kern0.5em {\left[{\left\langle \delta \boldsymbol{x}\right\rangle}_{\psi}\right]}^2=\left\langle \psi |{\widehat{\boldsymbol{x}}}^2|\psi \right\rangle -{\left\langle \psi |\widehat{\boldsymbol{x}}|\psi \right\rangle}^2;\kern0.5em \boldsymbol{x}=\boldsymbol{r},\boldsymbol{p}, $$

translates into the probability (modulus) and current (phase) components of a general wavefunction of Eq. (1). The explicit form of the momentum operator in position representation, $\widehat{\mathbf{p}}\left(\boldsymbol{r}\right)$= −i*ħ∇*, gives the following expression for the momentum factor,

$$ {\left[{\left\langle \delta \boldsymbol{p}\right\rangle}_{\psi}\right]}^2={\hbar}^2\left\{{\left[{\left\langle \delta \mathit{\nabla \phi}\right\rangle}_{\psi}\right]}^2+\raisebox{1ex}{$1$}\!\left/ \!\raisebox{-1ex}{$4$}\right.\ I\left[p\right]\right\}, $$

involving the classical Fisher information *I*[*p*]. Here, [〈*δ∇ϕ*〉_*ψ*_]^2^ = 〈(*∇ϕ*)^2^〉_*ψ*_ − [〈*∇ϕ*〉_*ψ*_]^2^ denotes the squared dispersion in the phase gradient,

$$ {\displaystyle \begin{array}{c}{\left[{\left\langle \delta \mathit{\nabla \phi}\right\rangle}_{\psi}\right]}^2=\int p{\left(\mathit{\nabla \phi}\right)}^2d\boldsymbol{r}-{\left[\int p\left(\mathit{\nabla \phi}\right)d\boldsymbol{r}\right]}^2=\raisebox{1ex}{$1$}\!\left/ \!\raisebox{-1ex}{$4$}\right.I\left[\phi \right]-{\left[\left(m/\hbar \right)\int \boldsymbol{j}d\boldsymbol{r}\right]}^2\\ {}={\left(m/\hbar \right)}^2\left\{{\left\langle {\boldsymbol{V}}^2\right\rangle}_{\psi }-{\left[{\left\langle \boldsymbol{V}\right\rangle}_{\psi}\right]}^2\right\}={\left(m/\hbar \right)}^2{\left[{\left\langle \delta \boldsymbol{V}\right\rangle}_{\psi}\right]}^2,\end{array}} $$

related to the average velocity, 〈***V***〉_*ψ*_ = ∫*p****V****d****r*** = ∫***j****d****r***, and the state nonclassical gradient information *I*[*ϕ*]. The squared dispersion of the particle momentum can thus be expressed in terms of the state result information content *I*[*ψ*],

$$ {\left[{\left\langle \delta \boldsymbol{p}\right\rangle}_{\psi}\right]}^2={\hbar}^2\ \left\{\raisebox{1ex}{$1$}\!\left/ \!\raisebox{-1ex}{$4$}\right.\ I\left[\psi \right]-{\left[\left(m/\hbar \right)\ {\left\langle \boldsymbol{V}\right\rangle}_{\psi}\right]}^2\right\}. $$

The Heisenberg uncertainty relation then reads:

$$ {\left[{\left\langle \delta \boldsymbol{r}\right\rangle}_{\psi}\right]}^2\left\{I\left[\psi \right]-{\left[\left(2m/\hbar \right)\ {\left\langle \boldsymbol{V}\right\rangle}_{\psi}\right]}^2\right\}>1. $$

## Entanglement entropy of molecular fragments

Consider a division of the electron density *ρ*(***r***) in a molecular system M = A—B containing *N* = ∫*ρ*(***r***)*d****r*** electrons in the fragment distributions ***ρ***(***r***) = {*ρ*_X_(***r***)} corresponding to the complementary subsystems A and B:

38 $$ \rho ={\rho}_{\mathrm{A}}+{\rho}_{\mathrm{B}},\kern0.5em \int {\rho}_{\mathrm{X}}d\boldsymbol{r}={N}_{\mathrm{X}},\kern0.5em {N}_{\mathrm{A}}+{N}_{\mathrm{B}}=N. $$

For example, these fragments can represent *atoms-in-molecules* (AIM) or their collections, functional groups, reactants, etc. This partition also applies to the associated division of the probability (shape factor) distribution *p*(***r***) = *ρ*(***r***)/*N*, ∫*p*(***r***)*d****r*** = 1,

39 $$ {\displaystyle \begin{array}{l}p={\rho}_{\mathrm{A}}/N+{\rho}_{\mathrm{B}}/N\equiv {\pi}_{\mathrm{A}}+{\pi}_{\mathrm{B}}\\ {}=\left({N}_{\mathrm{A}}/N\right)\ \left({\rho}_{\mathrm{A}}/{N}_{\mathrm{A}}\right)+\left({N}_{\mathrm{B}}/N\right)\ \left({\rho}_{\mathrm{B}}/{N}_{\mathrm{B}}\right)\equiv {P}_{\mathrm{A}}{p}_{\mathrm{A}}+{P}_{\mathrm{B}}{p}_{\mathrm{B}}.\end{array}} $$

Here the vectors ***π***(***r***) = {*π*_X_(***r***)} and ***p***(***r***) = {*p*_X_(***r***)} combine the fragment probability densities, unity normalized within the whole molecule and in individual subsystems, respectively,

40 $$ {\sum}_{\mathrm{X}}\int {\pi}_{\mathrm{X}}\left(\boldsymbol{r}\right)d\mathbf{r}={\sum}_{\mathrm{X}}{P}_{\mathrm{X}}=\int {p}_{\mathrm{X}}\left(\boldsymbol{r}\right)\kern0.5em d\boldsymbol{r}=1,\kern0.5em \mathrm{X}=\mathrm{A},\mathrm{B}, $$

while ***P*** = {*P*_X_ = ∫*π*_X_(***r***)*d****r*** = *N*_X_/*N*} contains the condensed probabilities of these constituent parts of M: *P*_A_ + *P*_B_ = 1.

These overall and subsystem probabilities generate the classical Shannon entropies reflecting the corresponding classical uncertainty descriptors. The global entropy of the molecule as a whole also defines the *total* entropy in division ***p***,

41 $$ S\left[p\right]=-\int p\left(\boldsymbol{r}\right)\ \ln p\left(\boldsymbol{r}\right)\ d\boldsymbol{r}\kern0.5em \equiv \kern0.5em {S}^{total}\left[\boldsymbol{p}\right]\kern0.5em \ge \kern0.5em 0, $$

while the component probabilities define the indeterminicity measures of the probability density in individual fragments:

42 $$ S\left[{p}_{\mathrm{X}}\right]=-\int {p}_{\mathrm{X}}\left(\boldsymbol{r}\right)\ln {p}_{\mathrm{X}}\left(\boldsymbol{r}\right)d\boldsymbol{r}\kern0.5em \equiv \kern0.5em {S}_{\mathrm{X}},\kern0.5em \mathrm{X}=\mathrm{A},\mathrm{B}. $$

Together they determine the *additive* entropy for this partitioning,

43 $$ {S}^{add.}\left[\boldsymbol{p}\right]={P}_{\mathrm{A}}\ {S}_{\mathrm{A}}+{P}_{\mathrm{B}}\ {S}_{\mathrm{B}}\ge 0, $$

and hence the associated *nonadditive* part of *S*^*total*^[***p***] = *S*[*p*]:

44 $$ {S}^{nadd.}\left[\boldsymbol{p}\right]={S}^{total}\left[\boldsymbol{p}\right]-{S}^{add.}\left[\boldsymbol{p}\right]. $$

The latter can also be expressed as the weighted average of entropy deficiencies [10,11] in the fragment probability densities relative to the molecular distribution,

45 $$ \Delta S\left[{p}_{\mathrm{X}}|p\right]=\int {p}_{\mathrm{X}}\left(\boldsymbol{r}\right)\ \ln \left[{p}_{\mathrm{X}}\left(\boldsymbol{r}\right)/p\left(\boldsymbol{r}\right)\right]\ d\boldsymbol{r}\ge 0, $$

measuring the corresponding information distances:

46 $$ {S}^{nadd.}\left[\boldsymbol{p}\right]={P}_{\mathrm{A}}\ \Delta S\left[{p}_{\mathrm{A}}|p\right]+{P}_{\mathrm{B}}\ \Delta S\left[{p}_{\mathrm{B}}|p\right]\ge 0. $$

The overall entropy in molecular electron density,

47 $$ S\left[\rho \right]=-\int \rho \left(\boldsymbol{r}\right)\ \ln \rho \left(\boldsymbol{r}\right)\ d\boldsymbol{r}=-{\sum}_{\mathrm{X}}\int {\rho}_{\mathrm{X}}\left(\boldsymbol{r}\right)\ \ln \rho \left(\boldsymbol{r}\right)\ d\boldsymbol{r}\equiv {S}^{total}\left[\boldsymbol{\rho} \right], $$

and its additive contribution,

48 $$ {S}^{add.}\left[\boldsymbol{\rho} \right]=-{\sum}_{\mathrm{X}}\int {\rho}_{\mathrm{X}}\left(\boldsymbol{r}\right)\ln {\rho}_{\mathrm{X}}\left(\boldsymbol{r}\right)d\boldsymbol{r}, $$

give the associated nonadditive component:

49 $$ {S}^{nadd.}\left[\boldsymbol{\rho} \right]={S}^{total}\left[\boldsymbol{\rho} \right]-{S}^{add.}\left[\boldsymbol{\rho} \right]={\sum}_{\mathrm{X}}\ \Delta S\left[{\rho}_{\mathrm{X}}|\rho \right]\equiv \Delta {S}^{add.}\left[\boldsymbol{\rho} |\rho \right]. $$

This nonadditive entropy thus measures the *additive* (molecularly-referenced) entropy deficiency in electron densities. It reflects the average information distance between the fragment and molecular densities. It can be used to describe the information similarity between constituent parts and the whole system: the smaller this missing information, the more the two fragments resemble the molecule [3, 34–36].

The IT descriptors of Eqs. (46) and (49) can be regarded as measures of the “binding” entropy in the mutually-*open* (entangled) subsystems for the specified molecular state Ψ yielding *ρ*, denoted as Ψ→*ρ*, in the *bonded* (molecular) composite system

50 $$ {\mathrm{M}}^{open}\left[\Psi \right]=\left({\mathrm{A}}^{\ast}\left[\Psi \right]\brokenvert {\mathrm{B}}^{\ast}\left[\Psi \right]\right)\equiv {\left({\mathrm{A}}^{\ast}\brokenvert {\mathrm{B}}^{\ast}\right)}_{\Psi}=\mathrm{M}, $$

since the additive entropies of Eqs. (43) and (48) characterize the mutually-*closed* (disentangled) subsystems in the *nonbonded* (“promolecular”) reference [27, 29, 31]

51 $$ {\mathrm{M}}^{closed}=\left({\mathrm{A}}^{+}\left[\rho \right]|{\mathrm{B}}^{+}\left[\rho \right]\right)\equiv \left({\mathrm{A}}^{+}|{\mathrm{B}}^{+}\right). $$

Above the mutual “bonding” and “non-bonding” character of the two fragments has been denoted by the vertical *broken*- and *solid*-lines, respectively, which separate these subsystems in the corresponding composite system.

One further observes that the molecularly-referenced *additive* information-distance of Eq. (49) supplemented by the local constraint of Eq. (38), of conserving the molecular electron density in the partition, gives the variational similarity criterion

52 $$ \delta \left\{\Delta {S}^{add.}\left[\boldsymbol{\rho} |\boldsymbol{\rho} \right]-{\sum}_{\mathrm{X}}\int \lambda \left(\boldsymbol{r}\right)\ {\rho}_{\mathrm{X}}\left(\boldsymbol{r}\right)\ d\boldsymbol{r}\right\}=0, $$

establishing the equal division of *ρ* between the two subsystems: *ρ*_X_ = *ρ*/2, X = A, B. It has been shown elsewhere [3, 34–36], however, that the information variational rule in terms of the *nonadditive* entropy-deficiency referenced to the densities ***ρ***^0^ = {*ρ*_X_^0^} of separate subsystems,

53 $$ \delta \left\{\Delta {S}^{nadd.}\left[\boldsymbol{\rho} |{\boldsymbol{\rho}}^0\right]-{\sum}_{\mathrm{X}}\int {\lambda}^0\left(\boldsymbol{r}\right)\ {\rho_{\mathrm{X}}}^0\left(\boldsymbol{r}\right)\ d\boldsymbol{r}\right\}=0, $$

generates the Hirshfeld [37] (“stockholder”) pieces of the molecular density:

54 $$ {\rho_{\mathrm{X}}}^{\mathrm{H}}={\rho_{\mathrm{X}}}^0\ \left(\rho /{\rho}^0\right)\equiv {\rho_{\mathrm{X}}}^0\ w\equiv \rho\ {d_{\mathrm{X}}}^0\kern1.5em \mathrm{or}\kern1.5em {d_{\mathrm{X}}}^0\equiv {\rho_{\mathrm{X}}}^0/{\rho}^0={\rho_{\mathrm{X}}}^{\mathrm{H}}/\rho \equiv {d_{\mathrm{X}}}^{\mathrm{H}}, $$

The optimum pieces of the molecular density can thus be regarded either as the local *molecular enhancement w*(***r***) *= ρ*(***r***)/*ρ*^0^(***r***) of the subsystem density *ρ*_X_^0^, or as the *promolecular share d*_X_^0^(***r***) = *ρ*_X_^0^(***r***)/*ρ*^0^(***r***) in the molecular density *ρ*(***r***). The promolecular distribution *ρ*^0^(***r***) = ∑_X_ *ρ*_X_^0^(***r***) is determined by the separate-fragment densities shifted to their actual positions in the molecule [3, 37]. Here,

55 $$ {\displaystyle \begin{array}{c}\Delta {S}^{nadd.}\left[\boldsymbol{\rho} |{\boldsymbol{\rho}}^0\right]=\Delta {S}^{total}\left[\boldsymbol{\rho} |{\boldsymbol{\rho}}^0\right]-\Delta {S}^{add.}\left[\boldsymbol{\rho} |{\boldsymbol{\rho}}^0\right]={\sum}_{\mathrm{X}}\int {\boldsymbol{\rho}}_{\mathrm{X}}\left(\boldsymbol{r}\right)\ln \left[{d_{\mathrm{X}}}^0\left(\boldsymbol{r}\right)/{d}_{\mathrm{X}}\left(\boldsymbol{r}\right)\right]d\boldsymbol{r}\\ {}={\sum}_{\mathrm{X}}\int {\boldsymbol{\rho}}_{\mathrm{X}}(r)\ln \left[w\left(\boldsymbol{r}\right)/{w}_{\mathrm{X}}(r)\right]d\boldsymbol{r},\kern1.25em {w}_{\mathrm{X}}\left(\boldsymbol{r}\right)={\rho}_{\mathrm{X}}\left(\boldsymbol{r}\right)/{\rho_{\mathrm{X}}}^0\left(\boldsymbol{r}\right),\\ {}\Delta {S}^{total}\left[\boldsymbol{\rho} |{\boldsymbol{\rho}}^0\right]=\Delta S\left[\rho |{\rho}^0\right],\kern1.25em \Delta {S}^{add.}\left[\boldsymbol{\rho} |{\boldsymbol{\rho}}^0\right]={\sum}_{\mathrm{X}}\Delta S\left[{\rho}_{\mathrm{X}}|{\rho_{\mathrm{X}}}^0\right].\end{array}} $$

The “stockholder” fragments thus exhibit the maximum information similarity to their isolated (promolecular) analogs, giving rise to the minimum of the relevant entropy-deficiency (missing-information) descriptors [3, 34–36]. A reference to Eq. (54) indicates that for this particular division scheme the nonadditive missing information of Eq. (39) exactly vanishes [3]:

56 $$ \Delta {S}^{nadd.}\ \left[{\boldsymbol{\rho}}^{\mathrm{H}}|{\boldsymbol{\rho}}^0\right]=0, $$

since *w*_X_^H^ = *w* and *d*_X_^H^ = *d*_X_^0^.

One further observes that expressing *ρ*_X_(***r***) in Δ*S*^*nadd.*^[***ρ***|***ρ***^0^] as *ρ*(***r***) *d*_X_(***r***) gives

57 $$ \Delta {S}^{nadd.}\left[\boldsymbol{\rho} |{\boldsymbol{\rho}}^0\right]=-\int \rho \left(\boldsymbol{r}\right)\left\{{\sum}_{\mathrm{X}}\ {d}_{\mathrm{X}}\left(\boldsymbol{r}\right)\ \ln \left[{d}_{\mathrm{X}}\left(\boldsymbol{r}\right)/{d_{\mathrm{X}}}^0\left(\boldsymbol{r}\right)\right]\right\}\ d\boldsymbol{r}=-\int \rho \left(\boldsymbol{r}\right)\ \Delta {S}^{add.}\left[\boldsymbol{d}\left(\boldsymbol{r}\right)|{\boldsymbol{d}}^0\left(\boldsymbol{r}\right)\right]\ d\boldsymbol{r}\le 0, $$

since both the local density *ρ*(***r***) and local additive information-distance Δ*S*^*add.*^[***d***(***r***)|***d***^0^(***r***)] are separately nonnegative. Therefore, the Hirshfeld subsystems also result from the *maximum* information principle for the nonadditive entropy deficiency:

58 $$ {\max}_{\boldsymbol{\rho}}\ \Delta {S}^{nadd.}\left[\boldsymbol{\rho} |{\boldsymbol{\rho}}^0\right]=\Delta {S}^{nadd.}\left[{\boldsymbol{\rho}}^{\mathrm{H}}|{\boldsymbol{\rho}}^0\right]=0. $$

The stockholder pieces of the molecular density thus exhibit the maximum nonadditivity relative to the promolecular distributions in the separate subsystems.

Let us now examine the entanglement entropy of molecular fragments in the *phase*-equilibrium state of Eq. (34), which maximizes the *resultant* entropy combining contributions from the modulus (probability) and phase (current) components [14–20]. Consider the *single*-electron (orbital) state *ψ*[*p*] = *R* exp(i*ϕ*), *ϕ* ≥ 0, with *p* = |*ψ*|^2^ = *R*^2^ = *ρ*. The overall global entropy contains the negative nonclassical (phase) contribution *S*[*ϕ*] proportional to the state average phase 〈*ϕ*〉. The equilibrium, *phase*-transformed state in M^*open*^[*ψ*→*p*],

59 $$ {\psi}_{eq.}\left[p\right]=\exp \left(\mathrm{i}{\phi}_{eq.}\left[p\right]\right)\ \psi \left[p\right]=R\ \exp \left(\mathrm{i}{\varPhi}_{eq.}\left[p\right]\right), $$

exhibits the resultant local phase

60 $$ {\varPhi}_{eq.}\left[p\right]=\phi \left[\psi \to p\right]+{\phi}_{eq.}\left[p\right]\equiv \phi \left[p\right]+{\phi}_{eq.}\left[p\right], $$

identified by the optimum “thermodynamic” phase-shift of Eq. (36) related to the system probability density:

61 $$ {\phi}_{eq.}\left[p;\boldsymbol{r}\right]=-\left(1/2\right)\ \ln p\left(\boldsymbol{r}\right)\equiv {\phi}_{\mathrm{M}}\left(\boldsymbol{r}\right). $$

In such an entangled state of subsystems, in the bonded (molecular) reference system ${\mathrm{M}}_{eq.}^{\ast }$ = (A^*^¦B^*^)_*eq.*_, the resultant phase *Φ*_*eq.*_[*p*] also characterizes each mutually-*open* (nonadditive) fragment X^*^ related to a common molecular “ancestor” state *ψ*[*p*]:

62 $$ {\phi}_{eq.}\left[p\right]={\phi}_{eq.}\left[{\mathrm{X}}^{\ast },p\right],\kern1.5em \mathrm{X}=\mathrm{A},\mathrm{B}. $$

The phase transformation of Eq. (59) generates the extra current contribution proportional to the probability gradient:

63 $$ \boldsymbol{j}\left[{\phi}_{eq.}\left[p\right]\right]=\left(\hbar /m\right)\ p\ \nabla {\phi}_{eq.}\left[p\right]=-\left(\hbar /2m\right)\ \nabla p\equiv {\boldsymbol{j}}_{eq.}\left[p\right]. $$

The equilibrium states of the mutually-*closed* (additive) subsystems {${\mathrm{X}}_{eq.}^{+}$} in ${\mathrm{M}}_{eq.}^{+}$= (${\mathrm{A}}_{eq.}^{+}$|${\mathrm{B}}_{eq.}^{+}$) are similarly described by the respective “thermodynamic” *phase*-shifts marking their own (internal) equilibria:

64 $$ {\phi}_{eq.}\left[{p}_{\mathrm{X}},\boldsymbol{r}\right]=-\left(1/2\right)\ \ln {p}_{\mathrm{X}}\left(\boldsymbol{r}\right)\equiv {\phi_{\mathrm{X}}}^{+}\left[{p}_{\mathrm{X}}\right],\kern1.5em \mathrm{X}=\mathrm{A},\mathrm{B}. $$

They generate the associated currents in molecular fragments,

65 $$ \left\{\ \boldsymbol{j}\left[{\phi_{\mathrm{X}}}^{+}\left[{p}_{\mathrm{X}}\right]\right]=\left(\hbar /m\right)\ {p}_{\mathrm{X}}\ \nabla {\phi_{\mathrm{X}}}^{+}\left[{p}_{\mathrm{X}}\right]=-\left(\hbar /2m\right)\ \nabla {p}_{\mathrm{X}}\equiv {{\boldsymbol{j}}_{\mathrm{X}}}^{+}\right\}, $$

and the resultant average current:

66 $$ \boldsymbol{j}\left({\mathrm{A}}^{+}|{\mathrm{B}}^{+}\right)={\sum}_{\mathrm{X}}\ {P}_{\mathrm{X}}\kern0.5em {{\boldsymbol{j}}_{\mathrm{X}}}^{+}=-\left(\hbar /2m\right)\ \nabla \left({\sum}_{\mathrm{X}}\ {P}_{\mathrm{X}}\ {p}_{\mathrm{X}}\right)=-\left(\hbar /2m\right)\ \nabla p\equiv {\boldsymbol{j}}^{+}. $$

In such a fragment resolution the overall entropy of M_*eq.*_=${\mathrm{M}}_{eq.}^{\ast }$ in the *phase*-equilibrium state *ψ*_*eq.*_[*p*] thus reads:

67 $$ {\displaystyle \begin{array}{c}{S}^{total}\left[{\psi}_{eq.}\left[p\right]\right]=S\left[{\mathrm{M}}_{eq.}^{\ast}\right]=S\left[{\psi}_{eq.}\left[p\right]\right]\\ {}=S\left[\psi \left[p\right]\right]+S\left[{\phi}_{eq.}\left(\mathrm{M}\right)\right]\\ {}=\left(S\left[p\right]+S\left[\phi \left[\psi \to p\right]\right]\right)+S\left[{\phi}_{\mathrm{M}}\left[p\right]\right]\\ {}=S\left[\phi \left[\psi \to p\right]\right],\end{array}} $$

since *S*[*p*] and

68 $$ S\left[{\phi}_{\mathrm{M}}\left[p\right]\right]=-2\int p\ {\phi}_{\mathrm{M}}\left[p\right]\ d\boldsymbol{r}=-S\left[p\right] $$

cancel each other. One also observes that the additive equilibrium component, describing the mutually nonbonded fragments in ${\mathrm{M}}_{eq.}^{+}$, exactly vanishes:

69 $$ {S}^{add.}\left[{\psi}_{eq.}\right]=S\left[{\mathrm{M}}_{eq.}^{+}\right]=-{\sum}_{\mathrm{X}}\int {p}_{\mathrm{X}}\left({\mathrm{lnp}}_{\mathrm{X}}+2{\phi_{\mathrm{X}}}^{+}\left[{p}_{\mathrm{X}}\right]\right)d\boldsymbol{r}=0. $$

Therefore, the nonadditive part of the resultant entropy in the *phase*-transformed, equilibrium state *ψ*_*eq.*_[*p*] is determined by the *phase*-entropy *S*[*ϕ*[*p*]], due to the phase of the original quantum state *ψ*[*p*], a common molecular “ancestor” of both the entangled subsystems in M:

70 $$ {S}^{nadd.}\left[{\psi}_{eq.}\right]={S}^{total}\left[{\psi}_{eq.}\right]-{S}^{add.}\left[{\psi}_{eq.}\right]=S\left[\phi \right]. $$

Indeed, the phenomenon of a quantum entanglement [28, 30, 31] for the given electron distribution in the molecule as a whole, has an exclusively *nonclassical* (phase/current) origin. This entropic descriptor of the fragment entanglement vanishes for the nondegenerate ground state *ψ = ψ*_0_, when the probability “degree-of-freedom” alone exactly identifies the molecular electronic state: *ϕ = ϕ*_0_ = 0, and hence ***j*** = ***j***_0_ = ***0***. This result is in accordance with the basic theorems of DFT [22], which predict that all physical properties in such a “classical” (real) state are determined by the molecular electron density alone. Therefore, when the molecular current exactly vanishes, a division of the molecular density, a “static” distribution of electrons, into the equilibrium fragment pieces amounts to a classical partition into a collection of *disentangled* subsystems, which is devoid of any phase (coherence) content.

## Affinities, fluxes, information production, and equilibrium

We now reexamine a related problem of the source term in the associated continuity equation for the resultant gradient information, generated by the coupled probability *p*(***r***, *t*) = *ψ*(***r***, *t*) ψ^*^(***r***, *t*) = *R*(***r***, *t*)^2^ and phase *ϕ*(***r***, *t*) = (2i)^−1^ln[*ψ*(***r***, *t*)/ψ^*^(***r***, *t*)] components of the molecular electronic state *ψ*(***r***, *t*). It can be approached using the standard treatment of irreversible thermodymamics [38].

Before addressing the problem of a production of the resultant information let us briefly examine the continuity of the classical gradient information *I*[*p*] of Eq. (18). Its functional derivative

71 $$ {F}_p^{class.}\left(\boldsymbol{r}\right)=\frac{\delta I\left[p\right]}{\delta p\left(\boldsymbol{r}\right)}={\left(\frac{\nabla p\left(\boldsymbol{r}\right)}{p\left(\boldsymbol{r}\right)}\right)}^2-2\left(\frac{\Delta p\left(\boldsymbol{r}\right)}{p\left(\boldsymbol{r}\right)}\right)=-4\left(\frac{\Delta R\left(\boldsymbol{r}\right)}{R\left(\boldsymbol{r}\right)}\right), $$

defines the local probability “intensity” of the classical information, which determines the functional differential, *dI*[*p*] = ∫ *F*_*p*_^*class.*^(***r***) *δp*(***r***) *d****r***, the Fisher information current, ***J***_*I*_^*class.*^(***r***) = *F*_*p*_^*class.*^(***r***) ***j***(***r***), its divergence:

72 $$ \nabla \cdot {{\boldsymbol{J}}_I}^{class.}\ \left(\boldsymbol{r}\right)=\nabla {F_p}^{class.}\left(\boldsymbol{r}\right)\cdot \boldsymbol{j}\left(\boldsymbol{r}\right)+{F_p}^{class.}\left(\boldsymbol{r}\right)\ \nabla \cdot \boldsymbol{j}\left(\boldsymbol{r}\right), $$

and derivative of the functional density:73Taking into account the probability continuity then gives the information source derivative:74This product of the classical probability “affinity” ***G***_*p*_^*class.*^(***r***) and “flux” ***j***(***r***) is thus seen to identically vanish for an ***r***-independent phase, i.e., the zero current, e.g., in the stationary state of Eq. (15). Turning now to the continuity problem of the resultant gradient information, one again recognizes the continuity relations of Eqs. (8) and (12) for the independent (instantaneous) probability and phase parameters of a general, complex wavefunction of Eq. (6),

75 $$ {\sigma}_p=\frac{d p}{d t}=\frac{\partial p}{\partial t}+\nabla \cdot \boldsymbol{j}=0\kern0.5em \mathrm{and}\kern0.5em {\sigma}_{\phi }=\frac{d\phi}{d t}=\frac{\partial \phi }{\partial t}+\nabla \cdot \boldsymbol{J}\kern0.5em \ne \kern0.5em 0, $$

expressing the associated dynamical equations resulting from SE:

76 $$ {\displaystyle \begin{array}{c}\partial p/\partial t=-\left(\hbar /m\right)\left]\ \right[\mathit{\nabla p}\cdot \mathit{\nabla \phi }+p\Delta \phi \Big]=-\boldsymbol{V}(t)\cdot \mathit{\nabla p}=-\left(\hbar /m\right){\left\langle \mathit{\nabla \phi}\right\rangle}_{\psi (t)}\cdot \mathit{\nabla p}\kern1.25em \mathrm{and}\\ {}\partial \phi /\partial t=\left[\hbar /(2m)\right]\ \left[{R}^{-1}\Delta R-{\left(\mathit{\nabla \phi}\right)}^2\right]-v/\hbar, \end{array}} $$

here ***j*** ≡ ***J***_*p*_ = *p****V*** denotes the probability current and ***J*** ≡ ***J***_*ϕ*_ = *ϕ****V*** stands for the *phase*-flux density, measuring the phase transported through unit area per unit time, and the phase source *σ*_*ϕ*_ is defined in Eq. (14). The local resultant intensities (per unit volume) ***F***(***r***) = [*F*_*p*_(***r***), *F*_*ϕ*_(***r***)] ≡ {*F*_*k*_(***r***)}, associated with the probability and phase components ***x***(***r***) = [*p*(***r***), *ϕ*(***r***)]} ≡ {*x*_*k*_(***r***)} and their currents **J**(***r***) = [***J***_*p*_(***r***), ***J***_*ϕ*_(***r***)]} ≡ {***J***_*k*_(***r***)}, are again given by the corresponding (partial) functional derivatives of *I*[*ψ*] = *I*[*p*, *ϕ*] with respect to these state parameters:

77 $$ {\displaystyle \begin{array}{l}{F}_p\left(\boldsymbol{r}\right)={\left(\frac{\partial I\left[p,\phi \right]}{\partial p\left(\boldsymbol{r}\right)}\right)}_{\phi }={\left(\frac{\nabla p\left(\boldsymbol{r}\right)}{p\left(\boldsymbol{r}\right)}\right)}^2-2\left(\frac{\Delta p\left(\boldsymbol{r}\right)}{p\left(\boldsymbol{r}\right)}\right)+4{\left[\nabla \phi \left(\boldsymbol{r}\right)\right]}^2=4\left({\left[\nabla \phi \left(\boldsymbol{r}\right)\right]}^2-\frac{\Delta R\left(\boldsymbol{r}\right)}{R\left(\boldsymbol{r}\right)}\right),\\ {}{F}_{\phi}\left(\boldsymbol{r}\right)={\left(\frac{\partial I\left[p,\phi \right]}{\partial \phi \left(\boldsymbol{r}\right)}\right)}_p=-8\left[\nabla p\left(\boldsymbol{r}\right)\cdot \nabla \phi \left(\boldsymbol{r}\right)+p(r)\Delta \phi \left(\boldsymbol{r}\right)\right].\end{array}} $$

They determine the differential of this resultant gradient information,78and suggest the associated information current

79 $$ {\boldsymbol{J}}_I\left(\boldsymbol{r}\right)={F}_p\left(\boldsymbol{r}\right)\ {\boldsymbol{J}}_p\left(\boldsymbol{r}\right)+{F}_{\phi}\left(\boldsymbol{r}\right)\ {\boldsymbol{J}}_{\phi}\left(\boldsymbol{r}\right)\equiv {\sum}_k\ {F}_k\left(\boldsymbol{r}\right)\kern0.5em {\boldsymbol{j}}_k\left(\boldsymbol{r}\right) $$

measuring the regional resultant information transported through unit area per unit time.

The rate of a local production of the resultant gradient information is given by the sum of the information leaving the region and the rate of the information source within this infinitesimal volume,80where:81Finally, using the continuity Eq. (75) identifies the source term of the resultant gradient information:82

The first term in the preceding equation is classical in character. As in irreversible thermodynamics [38] it combines products of regional *affinities* **G**(***r***) ≡ {***G***_*k*_(***r***) = *∇F*_*k*_(***r***)}, gradients of local intensities ***F***(***r***) ≡ {*F*_*k*_(***r***)}, and *fluxes* **J**(***r***) ≡ {***J***_*k*_(***r***)} associated with the state parameters ***x***(***r***) ≡ {*x*_*k*_(***r***)}. The former determine the information “forces” driving these conjugate flows,

83 $$ {\boldsymbol{G}}_k=\partial {\sigma}_I/\partial {\boldsymbol{J}}_k, $$

while the latter appear as information “responses” to these generalized perturbations:

84 $$ {\boldsymbol{J}}_k=\partial {\sigma}_I/\partial {\boldsymbol{G}}_k. $$

Due to the nonclassical (phase) contribution in the overall information measure, the rate of production of the resultant gradient information does not vanish for zero affinities:

85 $$ {\left.{\sigma}_I\left(\boldsymbol{r}\right)\right|}_{\mathbf{G}=\mathbf{0}}\kern0.5em ={F}_{\phi}\left(\boldsymbol{r}\right)\ {\sigma}_{\phi}\left(\boldsymbol{r}\right). $$

However, the equilibrium source of the gradient information vanishes for the zero phase intensity, *F*_*ϕ*_(***r***) = 0, when *∇ϕ*(***r***) = ***0***, e.g., in the stationary state of Eqs. (15) and (16), for *p*[*ψ*_*s*_] = *p*_*s*_(***r***) and *ϕ*[*ψ*_*s*_] = *ϕ*_*s*_(*t*), when *∇ϕ*_*s*_(*t*) = ***0*** and Δ*ϕ*_*s*_(*t*) = 0. In such eigenstates of the Hamiltonian operator one finds: {***J***_*p*_[*ψ*_*s*_] = ***0***, ***J***_*ϕ*_[*ψ*_*s*_] = ***J***_*ϕ*_ (*t*)}, {*F*_*p*_[*ψ*_*s*_] = *F*_*p*_^*class.*^, *F*_*ϕ*_[*ψ*_*s*_] = 0} and {***G***_*p*_[*ψ*_*s*_] = ***G***_*p*_^*class.*^, ***G***_*ϕ*_[*ψ*_*s*_] = ***0***}. The stationary state, corresponding to the sharply specified electronic energy of Eq. (16), thus exhibits a nonvanishing *probability*-intensity of Eq. (71),

86 $$ {F}_p\left[{\psi}_s\right]=-4\Delta {R}_s/{R}_s=8m\left({E}_s-v\right)/{\hbar}^2\kern1.5em \mathrm{or}\kern1.5em {\boldsymbol{G}}_p\left[{\psi}_s\right]=\left(8m/{\hbar}^2\right)\nabla v\ne \boldsymbol{0}, $$

and zero values of the *phase*-intensity and affinity: *F*_*ϕ*_[*ψ*_*s*_] = 0, ***G***_*ϕ*_[*ψ*_*s*_] = ***0***.

The vanishing production of the resultant gradient information in the stationary quantum states,

87 $$ {\sigma}_I\left[{\psi}_s\right]={\boldsymbol{G}}_p\left[{\psi}_s\right]\cdot {\boldsymbol{J}}_p\left[{\psi}_s\right]+{\boldsymbol{G}}_{\phi}\left[{\psi}_s\right]\cdot {\boldsymbol{J}}_{\phi}\left[{\psi}_s\right]+{F}_{\phi}\left[{\psi}_s\right]\ {\sigma}_{\phi}\left[{\psi}_s\right]=0, $$

identifies them as the system *information equilibra*. Thus, the stationary wavefunctions of molecular QM represent the zero production states of the overall gradient information. It should be observed, however, that contrary to the concept of equilibrium in irreversible thermodynamics [38], such IT equilibrium states do not correspond to the vanishing affinities **G** = **0**, since *σ*_*I*_[*ψ*_*s*_] vanishes due to ***J***_*p*_[*ψ*_*s*_] = ***G***_*ϕ*_[*ψ*_*s*_] = ***0*** and *F*_*ϕ*_[*ψ*_*s*_] = 0. A given displacement from this information equilibrium, specified by the applied “forces” **G**, triggers probability and phase flows with each flux depending on all affinities and all intensities: ***J***_*k*_ = ***J***_*k*_(**G**, ***F***). One recalls that in ordinary thermodynamics [38] each flux depends most strongly on its own affinity and it is also known to vanish as these affinities vanish, so one can expand the currents in powers of the affinities with no constant term.

The intensity and affinity concepts corresponding to the *phase equilibrium* of Eq. (61) are also of interest:

88 $$ {F_{\phi}}^{eq.}=-\nabla \cdot \left(8p\nabla {\phi}_{eq.}\right)=4\Delta p\kern1.5em \mathrm{and}\kern1.5em {{\boldsymbol{G}}_{\phi}}^{eq.}\kern0.5em =4{\nabla}^3p. $$

The former is thus related to the Fick’s diffusion equation [28] (see Eq. (63)]:

89 $$ {\left(\partial p/\partial t\right)}_{diff.}=-\nabla \cdot {\boldsymbol{j}}_{eq.}\left[p\right]=\left[\hbar /(2m)\right]\ \Delta p\kern.3em \equiv \kern.3em D\ \Delta p, $$

which formally identifies the electron diffusion coefficient *D* = *ħ*/(2 *m*) and the associated current for this migration:

90 $$ {\boldsymbol{j}}_{diff}.=-D\ \nabla p={\boldsymbol{j}}_{eq.}\left[p\right]. $$

## Dynamics of resultant entropy/information descriptors

Let us now reexamine a temporal evolution of the overall measures of the information and entropy content in the specified molecular quantum state |*ψ*(*t*)〉. One recalls that the average energy *E*[*ψ*(*t*)] of an isolated molecular system,

91 $$ {\displaystyle \begin{array}{l}E\left[\psi (t)\right]={\left\langle E\right\rangle}_{\psi (t)}=\left\langle \psi (t)|\widehat{\mathrm{H}}|\psi (t)\right\rangle =T\left[\psi (t)\left]+V\right[\psi (t)\right]\equiv E(t),\\ {}T\left[\psi (t)\right]=\left\langle \psi (t)|\widehat{\mathrm{T}}|\psi (t)\right\rangle \equiv {\left\langle T\right\rangle}_{\psi (t)}\equiv T(t),\\ {}V\left[\psi (t)\right]=\left\langle \psi (t)\left|v\right|\psi (t)\right\rangle \equiv {\left\langle V\right\rangle}_{\psi (t)}=\int p\left(\boldsymbol{r}\right)v\left(\boldsymbol{r}\right)d\boldsymbol{r}\equiv V(t),\end{array}} $$

remains conserved in time:

92 $$ dE(t)/ dt=\left(\mathrm{i}/\hbar \right)\left\langle \psi (t)\left|\left[\widehat{\mathrm{H}},\widehat{\mathrm{H}}\right]\right|\psi (t)\right\rangle =0. $$

One similarly explores time dependency of overall measures of the complementary entropy/information descriptors: the expectation values of the state complex entropy,

93 $$ H\left[\psi (t)\right]={\left\langle H\right\rangle}_{\psi (t)}=\left\langle \psi (t)\left|\widehat{\mathrm{S}}(t)\right|\psi (t)\right\rangle =\int p\left(\boldsymbol{r},t\right)\left[-2\ln \psi \left(\boldsymbol{r},t\right)\right]d\boldsymbol{r}=S\left[p(t)\right]+\mathrm{i}S\left[\phi (t)\right]\kern.3em \equiv \kern.3em H(t), $$

and its resultant gradient information:

94 $$ I\left[\psi (t)\right]={\left\langle I\right\rangle}_{\psi (t)}=\left\langle \psi (t)\left|\widehat{\mathrm{I}}\right|\psi (t)\right\rangle =I\left[p(t)\right]+I\left[\phi (t)\left]=I\right[p(t)\left]+I\right[\boldsymbol{j}(t)\right]\kern.3em \equiv \kern.3em I(t). $$

One first observes that a direct differentiation of the complex-entropy functional *H*(*t*) = ∫ψ^*^(***r***, *t*) *S*_*ψ*_(***r***, *t*)*ψ*(***r***, *t*) *d****r*** = ∫*p*(***r***, *t*) *S*_*ψ*_(***r***, *t*) *d****r*** gives:

95 $$ {\displaystyle \begin{array}{l}{\sigma}_H(t)\kern.3em \equiv \kern.3em dH(t)/ dt=\int {S}_{\psi}\left(\boldsymbol{r},t\right)\left[\partial p\left(\boldsymbol{r},t\right)/\partial t\right]d\boldsymbol{r}+\int p\left(\boldsymbol{r},t\right)\left[\partial S\psi \left(\boldsymbol{r},t\right)/\partial t\right]d\boldsymbol{r}\\ {}=2\int \left[\ln \psi \left(\boldsymbol{r},t\right)\right]\nabla \cdot \boldsymbol{j}\left(\boldsymbol{r},t\right)d\boldsymbol{r}-2\int {\psi}^{\ast}\left(\boldsymbol{r},t\right)\left[\partial \psi \left(\boldsymbol{r},t\right)/\partial t\right]d\boldsymbol{r}\\ {}=\int \left[\ln p\left(\boldsymbol{r},t\right)+2 i\phi \left(\boldsymbol{r},t\right)\right]\nabla \cdot \boldsymbol{j}\left(\boldsymbol{r},t\right)d\boldsymbol{r}+\left(2i/\hbar \right)\int {\psi}^{\ast}\left(\boldsymbol{r},t\right)\widehat{\mathrm{H}}\left(\boldsymbol{r}\right)\psi \left(\boldsymbol{r},t\right)d\boldsymbol{r}\\ {}=\left(\hbar /m\right)\int \left[\ln p\left(\boldsymbol{r},t\right)+2 i\phi \left(\boldsymbol{r},t\right)\right]\left[\mathit{\nabla p}\left(\boldsymbol{r},t\right)\cdot \nabla \phi \left(\boldsymbol{r},t\right)+p\left(\boldsymbol{r},t\right)\Delta \phi \left(\boldsymbol{r},t\right)\right]d\boldsymbol{r}+2\mathrm{i}\omega (t).\end{array}} $$

where *ω*(*t*) = *E*(*t*)/*ħ*. Therefore, this complex derivative exhibits the following real and imaginary components:

96 $$ {\displaystyle \begin{array}{c}\operatorname{Re}\left( dH\left[p,\phi \right]/ dt\right)= dS\left[p\right]/ dt=\int \left(\ln \kern0.1em p\right)\mathit{\nabla}\cdot \boldsymbol{j}\kern0.5em d\boldsymbol{r},\\ {}\operatorname{Im}\left( dH\left[p,\phi \right]/ dt\right)= dS\left[\phi \right]/ dt=2\left[\int \phi \mathit{\nabla}\cdot \boldsymbol{j}\kern0.5em d\boldsymbol{r}+\omega (t)\right].\end{array}} $$

A reference to the real part *dS*[*p*]/*dt* directly shows that it has a nonclassical (current) genesis, vanishing in the classical limit, for *ϕ* = 0 or ***j*** = ***0***, when *S*[*ϕ*] = 0. Indeed it is the probability current ***j*** that drives changes in the probability distribution [see Eq. (5b)]. Clearly, the imaginary part of the derivative results alone from the nonclassical phase component *S*[*ϕ*] of the complex entropy. One thus finally concludes that the time evolution of the entire complex entropy exhibits the phase/current origin.

This result can also be demonstrated via the differentiation of the expectation value *H*(*t*) = 〈*ψ*(*t*)|$\widehat{\mathrm{S}}(t)$|*ψ*(*t*)〉 and by subsequently using SE and probability continuity:97where $\left[\widehat{\mathrm{H}},\widehat{\mathrm{S}}\right]$ = $\left[\widehat{\mathrm{T}},\widehat{\mathrm{S}}\right]$ = (*ħ*^2^/*m*) [*∇* ^2^, ln*ψ*] = (*ħ*^2^/*m*){*∇* ⋅ [*∇*, ln*ψ*] + [*∇*, ln*ψ*] ⋅ *∇*} and

98 $$ \left[\nabla, \ln \psi \right]=\mathrm{\nabla ln}\psi =\mathit{\nabla}\ln R+ i\mathit{\nabla \phi }=\raisebox{1ex}{$1$}\!\left/ \!\raisebox{-1ex}{$2$}\right.\mathrm{\nabla ln}p+\mathrm{i}\mathrm{\nabla}\phi ={R}^{-1}\nabla R+\mathrm{i}\mathrm{\nabla}\phi . $$

In the Schrödinger dynamical picture, the time change of the resultant gradient information, the operator of which does not depend on time explicitly, $\widehat{\mathrm{I}}\left(\boldsymbol{r}\right)$= −4*∇*^2^ = (8 *m*/*ħ*^2^)$\widehat{\mathrm{T}}\left(\boldsymbol{r}\right)$, results solely from the time dependence of the system state vector itself. Therefore, the time derivative of the average Fisher-type gradient (determinicity) information is generated by the expectation value of the commutator $\left[\widehat{\mathrm{H}},\widehat{\mathrm{I}}\right]$ alone,

99 $$ dI(t)/ dt=\left(\mathrm{i}/\hbar \right)\left\langle \psi (t)\left|\left[\widehat{\mathrm{H}},\widehat{\mathrm{I}}\right]\right|\psi (t)\right\rangle \kern.3em \equiv \kern.3em \left(\mathrm{i}/\hbar \right){\left\langle \left[\widehat{\mathrm{H}},\widehat{\mathrm{I}}\right]\right\rangle}_{\psi (t)}, $$

100 $$ \left[\widehat{\mathrm{H}},\widehat{\mathrm{I}}\right]=\left[v,\widehat{\mathrm{I}}\right]=4\left[{\nabla}^2,v\right]=4\left\{\left[\nabla, v\right]\cdot \nabla +\nabla \cdot \left[\nabla, v\right]\right\},\kern0.5em \left[\nabla, v\right]=\nabla v, $$

and the integration by parts implies 〈*ψ*|*∇ψ*〉 = − 〈*∇ψ*|*ψ*〉 ≡ 〈*∇*^†^*ψ*|*ψ*〉 or *∇*^†^ = −*∇*. Hence, the time derivative of the overall gradient information reads:

101 $$ {\displaystyle \begin{array}{l}{\sigma}_I(t)\kern.3em \equiv \kern.3em dI(t)/ dt=\left(4\mathrm{i}/\hbar \right)\left\{\left\langle \psi |\nabla v\cdot |\nabla \psi \right\rangle -\left\langle \nabla \psi |\cdot \nabla v|\psi \right\rangle \right\}\\ {}=-\left(8/\hbar \right) \operatorname {Im}\left\langle \psi (t)|\nabla v\cdot |\nabla \psi (t)\right\rangle =-\left(8/\hbar \right) \operatorname {Im}\left[\int \psi {\left(\boldsymbol{r},t\right)}^{\ast}\nabla v\left(\boldsymbol{r}\right)\cdot \nabla \psi \left(\boldsymbol{r},t\right)d\boldsymbol{r}\right]\\ {}=-\left(8/\hbar \right)\int p\nabla \phi \cdot \nabla v\kern0.5em d\boldsymbol{r}=-\left(8m/{\hbar}^2\right)\int \boldsymbol{j}\cdot \nabla v\kern0.5em d\boldsymbol{r}.\end{array}} $$

Again, this total time-derivative of the resultant gradient information is seen to be determined by the current content of the molecular electronic state. Therefore, it identically vanishes for the zero current density everywhere for *ϕ*(***r***) = 0, thus again confirming its nonclassical origin.

## Conclusions

The IT approach has proven its utility in a variety of molecular scenarios, e.g., [39–42]. In this analysis we examined mutual relations between densities of the classical and nonclassical components of the resultant information/entropy measures, combining the probability contributions of Fisher or Shannon and their associated phase/current supplements. For example, the complex (“vector”) entropy approach combines the classical (real) and nonclassical (imaginary) contributions due to the state probability (wavefunction modulus) and current (wavefunction phase), respectively. Such generalized entropic concepts allow one to distinguish the information content of states generating the same electron density but differing in their phase/current composition. They also allow a more precise information-theoretic description of the bonding status of molecular fragments. The IT principles using the resultant quantum descriptors of the entropy/information content in electronic states have also been used to determine the phase and information equilibria in molecules and their constituent parts [12–18]. The phase aspect of molecular states is also vital for the quantum (amplitude) communications between atoms in molecules [2–5, 42], which determine entropic descriptors of the chemical bond multiplicities and their covalent/ionic composition.

The need for the nonclassical (phase/current) supplements of the classical (probability) measures of the information content in molecular states has been stressed. The electron density distribution determines a static facet of the molecular structure, while the current distribution describes its dynamic aspect. Both these structural manifestations contribute to the overall information content of the generally complex electronic states of molecular systems, reflected by resultant IT concepts. The total time derivatives of such entropic descriptors of electronic states have been examined. These time dependencies have been established via the Schrödinger equation and the dynamics/continuity it implies for the classical and nonclassical degrees-of-freedom of complex wavefunctions. The nonclassical origin of the net temporal changes in the overall entropy/information quantities have been demonstrated. Thus, in real electronic states, exhibiting the vanishing local phase and current, the time derivatives of the resultant gradient information and global entropy exactly vanish.

Although, for simplicity reasons, we have assumed the *one*-electron case, the modulus (density), and the phase (current) aspects of general electronic states can be similarly separated [2] using the Harriman-Zumbach-Maschke (HZM) construction [43, 44] of Slater determinants yielding the specified electron density. The present *single*-electron development can then be naturally generalized into *many*-electron states of atomic and molecular systems. One observes that in HZM construction the common modulus part of *N* occupied (orthonormal) *equidensity orbitals* (EO), for the given ground-state density *ρ*, ***ψ***[*ρ*] = {*ψ*_*w*_[*p*]}, reflects the molecular probability distribution *p*(***r***) = *ρ*(***r***)/*N*, and so do the system optimum (orthonormal) Kohn-Sham (KS) [23] orbitals: ***φ***[*ρ*] = {*φ*_*t*_[*p*]}. In fact the two sets constitute the *equivalent* sets of spin-orbitals linked by the unitary transformation and generating identical Slater determinants, Ψ(*N*) ≡ det(***ψ***[*ρ*]) = det(***φ***[*ρ*]) ≡ Φ(*N*), and hence the invariant overall entropy or information measures.

For example, the expectation value of the *N*-electron operator for the overall gradient information,  = ∑_*i*_$\widehat{\mathrm{I}}(i)$, {$\widehat{\mathrm{I}}(i)$= −4Δ_*i*_}, reads:102where *T*_*s*_(*N*) stands for the kinetic energy of noninteracting electrons in the KS limit [23]. Thus, the amount of resultant gradient information in the occupied HZM orbitals derived from the optimum molecular distribution of electrons equals to that contained in orbitals describing the separable KS system corresponding to the same ground-state density. Notice, however, that the proportions between the classical (probability) and nonclassical (phase) information contributions vary with different partitions of the molecular density into orbital components.

In the DFT-based theory of chemical reactivity one distinguishes between several hypothetical stages involving either the mutually bonded (entangled) or nonbonded (disentangled) states of reactants for the same electron distribution in constituent subsystems. These two categories are discerned by the phase aspect of the quantum entanglement between such molecular fragments, e.g., [2, 27]. We have identified the classical entropic descriptor of this phenomenon, the nonadditive global entropy, which has been interpreted as the partition additive entropy-deficiency measuring the average information-distance between the fragment and molecular densities. The equilibrium phases and currents of reactants can be related to the relevant electron densities using the entropic principles of the quantum IT. This generalized approach deepens our understanding of the molecular/promolecular promotions of the constituent molecular fragments and provides a more precise framework for describing the hypothetical stages invoked in the theory of the chemical bond and reactivity.

The phenomenological apparatus of irreversible thermodynamics [38] also provides an attractive basis for an entropic representation of elementary molecular processes [2]. In this analysis we approached anew the problem of productions of the overall measures of information/entropy, which take into account both the modulus and phase components of complex wavefunctions. We introduced the relevant intensity and affinity conjugates of both the probability and phase fluxes, which together define a local production of the state information content. The nonvanishing local phase source has been identified, giving rise to the nonclassical contribution in the local production of the resultant entropy/information content. It has been argued that the criterion of the vanishing production of the gradient information identifies the stationary states of molecular QM as the system information equilibria. The local information-source has also been interpreted “thermodynamically”, by separating a classical summation over products of affinities (“perturbations”) and fluxes (“responses”) associated with the probability and phase/current degrees-of-freedom of molecular states. Since spontaneous flows driven by displacements in the given information affinity should act in a direction to restore the equilibrium, these elementary products should be negative; thus, decreasing the state information (determinicity) level, and hence increasing the state entropy (uncertainty, indeterminicity) content. This suggests a positive entropy production, and hence a negative information source.

To conclude this analysis, let us briefly comment on the resultant entropy concepts containing an explicit phase contribution [Eqs. (24) and (29)], the Shannon entropy of electron probability distribution [Eq. (19)], and von Neumann’s (vN) ensemble average entropy [45] contained in the density operator. The latter is defined as mathematical trace involving the density operator $\widehat{\uprho}$ of the statistical mixture in question,

103 $$ {S}_{\mathrm{vN}}\left[\widehat{\uprho}\right]=-\mathrm{tr}\left(\widehat{\uprho}\ln \widehat{\uprho}\right) $$

expressed in terms of its eigenvectors {|*ψ*_*j*_〉} and eigenvalues (probabilities) {|*η*_*j*_〉}:

104 $$ \widehat{\uprho}\left|{\psi}_i\right\rangle ={\eta}_i\left|{\psi}_i\right\rangle, \kern0.5em \widehat{\uprho}={\sum}_j\left|{\psi}_j\right\rangle {\eta}_j\left\langle {\psi}_j\right|. $$

It generates the information entropy contained in the ensemble state probabilities:

105 $$ {S}_{\mathrm{vN}}\left[\widehat{\uprho}\right]=-{\sum}_j{\eta}_j\ln {\eta}_j. $$

This measure identically vanishes for the pure quantum state |*ψ*〉, when ${\widehat{\uprho}}_{\psi }$ = |*ψ*〉 〈*ψ*| and *η*_*ψ*_ = 1: *S*_vN_[${\widehat{\uprho}}_{\psi }$] = 0. The (idempotent) density operator ${\widehat{\uprho}}_{\psi }$ then determines the Hermitian density matrix in position representation,

106 $$ \gamma \left(\boldsymbol{r},{\boldsymbol{r}}^{'}\right)=\left\langle \boldsymbol{r}\left|{\widehat{\uprho}}_{\psi}\right|{\boldsymbol{r}}^{'}\right\rangle =\psi \left(\boldsymbol{r}\right){\psi}^{\ast}\left({\boldsymbol{r}}^{'}\right),\kern0.5em \gamma \left(\boldsymbol{r},\boldsymbol{r}\right)=p\left(\boldsymbol{r}\right), $$

in terms of which the Shannon entropy *S*[*p*] contained in the probability density *p*(***r***) reads:

107 $$ S\left[p\right]=S\left[{\widehat{\uprho}}_{\psi}\right]=\int d\boldsymbol{r}\int d{\boldsymbol{r}}^{'}\kern0.5em \gamma \left(\boldsymbol{r},{\boldsymbol{r}}^{'}\right)\kern0.5em \left[-\delta \left(\boldsymbol{r},{\boldsymbol{r}}^{'}\right)\ln \kern0.5em \gamma \left({\boldsymbol{r}}^{'},\boldsymbol{r}\right)\right]\kern.3em \equiv \kern.3em \mathrm{tr}\left[{\widehat{\uprho}}_{\psi }{\widehat{\mathrm{S}}}_{\gamma}\right]. $$

Indeed, in this pure-state case the above “ensemble” average measure reduces to the expectation value in state |*ψ*〉, *S*[*p*] = 〈*ψ*|${\widehat{\mathrm{S}}}_{\psi}^{class.}$|*ψ*〉, of the classical (Hermitian) entropy operator [see Eq. (22)],

108 $$ {\widehat{\mathrm{S}}}_{\psi}^{class.}=\frac{1}{2}\left({\widehat{\mathrm{S}}}_{\psi }+{\widehat{\mathrm{S}}}_{\psi}^{\ast}\right)=\operatorname{Re}\left({\widehat{\mathrm{S}}}_{\psi}\right)=-\ln p. $$

Therefore, in the familiar Shannon entropy of classical IT, which reconstructs the ensemble-average measure of von Neuman’s quantum entropy in density matrix, the phase/current information terms of the complex entropies *S*[*ψ*] and *S*[ψ^*^] = *S*[*ψ*]^*^ cancel out, as indeed expected of the expectation value of the Hermitian operator ${\widehat{\mathrm{S}}}_{\psi}^{class.}$. One recalls that in QM one represents the physical properties by the associated (linear) Hermitian operators. However, the information entropy is neither an observable, determined in an experiment, nor is it linear in the underlying probability argument. Therefore, attributing to the overall quantum entropy content in the specified quantum state a non-Hermitian operator is an admissible, workable proposition, capable of a unique phase characterization of the entangled molecular subsystems [31].
